# Successful secondary thromboprophylaxis with direct oral anticoagulants for a patient with catastrophic antiphospholipid syndrome: A case report

**DOI:** 10.1097/MD.0000000000020811

**Published:** 2020-06-26

**Authors:** Nhan Hieu Dinh, Suzanne Monivong Cheanh Beaupha

**Affiliations:** a Department of Internal Medicine; b Department of Pharmacology; c Department of Hematology, University of Medicine and Pharmacy at Ho Chi Minh City, Ho Chi Minh City, Vietnam.

**Keywords:** antiphospholipid syndrome, CAPS, catastrophic antiphospholipid syndrome, DOACs

## Abstract

**Rationale:**

Catastrophic antiphospholipid syndrome (CAPS) is a rare, life-threatening condition of antiphospholipid syndrome (APS). Treatment and management of CAPS remain challenging and the mortality rate is approximately 50% among cases. We describe a successfully treated case of a CAPS patient who had undergone massive bowel resection due to obstruction of superior mesenteric artery.

**Patient concerns:**

A 40-year-old male patient was admitted to our hospital with acute abdominal pain, melena, and a history of deep vein thrombosis in both legs for over 10 years, there was no previous diagnosis of APS.

**Diagnosis:**

The patient was diagnosed as CAPS with bowel necrosis due to obstruction of superior mesenteric artery based on the presence of antiphospholipid antibodies, computed tomography scan, and histopathological examination.

**Interventions:**

Emergency surgery was performed to remove approximately 6 meters of the necrotic small intestine, of which the length of the remaining small intestine was 40 cm from the duodenum and 80 cm from the ileocaecal valve. Anticoagulants were prescribed with low molecular weight heparin. After discharging, APS was managed with direct oral anticoagulants (DOACs) for secondary thromboprophylaxis because the patient was unable to reach target International Normalized Ratio (INR) with vitamin K antagonists (VKAs).

**Outcomes:**

During 24 months of follow-up until now, the patient did not develop new thrombosis or relapse CAPS and his state remained stable.

**Lessons:**

While VKAs is amongst the most important and fundamental treatment, physicians should be aware that VKAs are absorbed via the small intestine. For CAPS cases who had undergone massive bowel resection, DOACs is a reasonable alternative which has been found to be as safe and effective as VKAs in terms of thrombosis prevention.

## 1 Introduction

Antiphospholipid syndrome (APS) is an autoimmune disorder characterized by arterial and venous thrombosis caused by antiphospholipid antibodies.^[1]^ Catastrophic antiphospholipid syndrome (CAPS) was first described by Asherson in 1992^[2]^ (therefore also known as Asherson syndrome) as a rare life-threatening condition of antiphospholipid syndrome with multiple intravascular thrombosis that occurred within 1 week leading to organ ischemia and multiple organ failure. This rare condition is often overlooked since most patients are hospitalized in emergency condition with multiple organ dysfunction syndrome. Treatment and management of CAPS remain challenging and the mortality rate is approximately 50% among cases.^[3,4]^ In this paper, we describe a case of a 40-year-old male CAPS patient who had undergone massive bowel resection. After 2 years treated with direct oral anticoagulants (DOACs), the patient experienced good clinical condition. The patient has provided informed consent for publication of the case.

## 2 Case presentation

A 40-year-old Vietnamese male patient was admitted to our hospital due to acute abdominal pain for the last 2 days. On day 1, while driving, the patient experienced sudden epigastric pain and was admitted to a private clinic and prescribed an endoscopy which had shown normal results, treatment is unknown but the pain was not relieved. On the next day, the patient continuously had epigastric pain, accompanied by nausea and melena. He was admitted to a district hospital with a diagnosis of bowel necrosis due to obstruction of superior mesenteric artery and transferred to a tertiary hospital on the same day. He has a history of deep vein thrombosis in both legs for over 10 years being treated with Acenocoumarol (adjusted dose according to international normalized ratio [INR]) for 2 years combined with Daflon, but has discontinued Acenocoumarol for the last 8 years. In terms of family history, he has a sister diagnosed with cerebral stroke and hypertension at 49-year old.

On clinical examination at admission, the patient was alert and oriented but significant pale skin and mucous membranes with toxic appearance were noticed. His pulse rate was 160 bpm, blood pressure was 95/70 mm Hg (under nor-adrenalin infusion), respiratory rate was 24 times per minute and temperature was 37^o^C. Urine = 0 mL. His height was 162 cm, weight was 80 kg, BMI of 32 indicated obese. He has significant continuous melena. A nasogastric tube was inserted for drainage and decompression to prepare for a surgery on the lower gastrointestinal tract. Green-colored gastric residual indicated a low probability of upper gastrointestinal bleeding. With pain throughout the abdomen, positive signs of abdominal resistance were recorded.

Laboratory findings revealed disseminated intravascular coagulation with low platelet 64,000/mm^3^, prothrombin time 54.9 s, INR 1.91 (under treatment with Lovenox), aPPT 45.8 s, fibrinogen 4.67 g/L, protein C 71%, protein S 51%. Result of quantitative analysis of factor Leiden V factor was 2.61. Diagnosis of systemic lupus erythematosus (SLE) was excluded with antinuclear antibody negative, anti-dsDNA 0 IU/mL, SLE (LE cell) negative.

**Figure 1:**
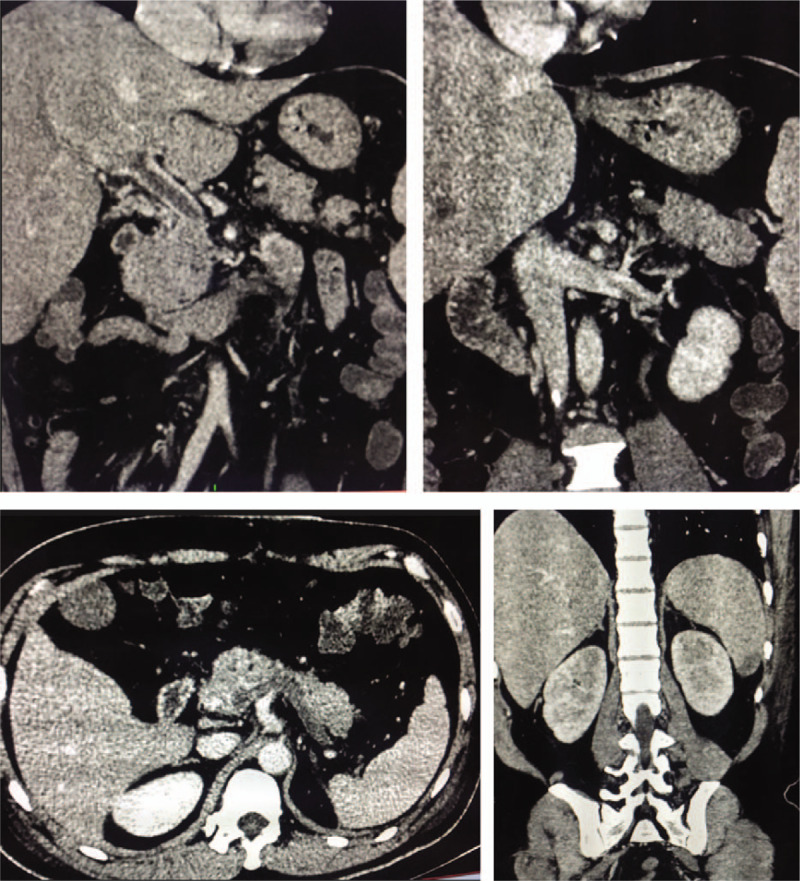
Results from abdominal CT scan with contrast: thrombosis of the portal vein, superior mesenteric vein, and injury of lower extremity of spleen. CT = computed tomography.

Ultrasonography of the lower extremity veins revealed partial occlusion of lower extremity veins in both legs from popliteal vein to external iliac vein and great saphenous veins close to the saphenofemoral junction. Abdominal computed tomography scan with contrast demonstrated a low attenuation area accounted for one-third under spleen, thought to be infarction, almost complete occlusion of superior mesenteric artery, partial occlusion of the superior mesenteric vein, main portal vein, parts of left portal vein close to the splitting site, thrombosis of left renal vein, and complete occlusion of spleen vein, right portal vein, inferior vena cava collapse (Fig. 1). Histopathological examination of 2 intestinal segments of about 1 cm long revealed ulcerative colitis, hemorrhage from mucosa to serosa. Antiphospholipid tests reported positive lupus anticoagulant. After receiving the examination results and considering the acute occurrence, rapid progression, and multiple venous thrombosis in this patient, we suspected that this case is CAPS. The patient had fulfilled classification criteria for CAPS,^[5]^ including vascular thrombosis of at least 3 organs, systems, or tissues (including lower extremity veins, superior mesenteric vein, spleen vein, and renal vein); symptoms occur within 1 week; histopathological evidence of intravascular thrombosis at least in 1 organ, or tissue and presence of antiphospholipid antibody (lupus anticoagulant) at 2 examination every 12 weeks. For the antibody criterion, examination was repeated after 4 months and 1 year to confirm the diagnosis (Table 1).

**Table 1:**
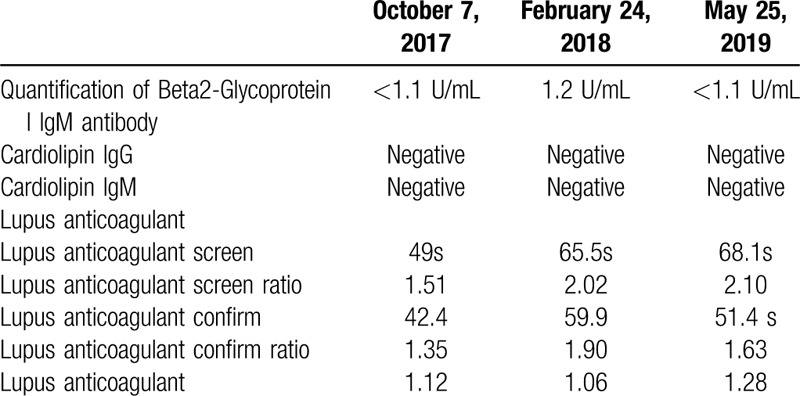
Tests to diagnose antiphospholipid syndrome.

**Figure 2:**
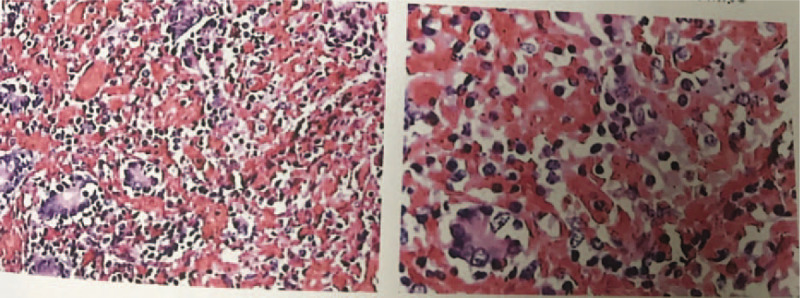
Histopathology results revealed hemorrhage from mucosa to intestinal serosa.

Emergency surgery was performed immediately to remove a total of approximately 6 m of the necrotic small intestine (Fig. 2), the remaining small intestine was 40 cm after the duodenum and 80 cm before the ileocaecal valve. Anticoagulants were prescribed with low molecular weight heparin (Lovenox 0.6 mL × 2 subcutaneous injection every 12 hours). When clinical outcomes are stable he was switched to Acenocoumarol (Sintrom) with dose adjusted in accordance with INR value (target INR from 2 to 3). The patient was discharged after 3 months. At revisit, his condition was stable but INR did not reach the target value and was continuously unable to be controlled after 3 follow-up revisits, so he was switched to DOACs, specifically dabigatran (Pradaxa) 110 mg twice daily or rivaroxaban (Xarelto) 15 mg per day (depending on the drug available under health insurance program). For the last 12 months until now, the patient has been prescribed rivaroxaban 15 mg per day. During 24 months of follow-up, the patient did not develop new thrombosis or relapse CAPS and his state remained stable.

## 3 Discussion and conclusion

Management of CAPS with bowel necrosis is challenging. Although studies have highlighted the importance of clinical suspicion to immediately provide proper treatment that can improve survival, the diagnosis is difficult due to the rareness of this disease.^[6–8]^ Treatment for the disease remains elusive since a high mortality rate was consistently reported, including a 67-year-old woman who died 15 days after admission^[6]^ and a 50-year-old man who died 22 days after admission.^[8]^ Recently, a 29-year-old CAPS patient survived after jejunal excision and duodeno-ileostomy was reported by Lee ^[7]^ Similar to our case, this patient was managed by low molecular weight heparin followed by DOACs after discharging, but the effectiveness of thromboprophylaxis was not mentioned probably because the follow-up time was not sufficiently long enough.^[7]^ Another problem that needs to be taken into consideration is the impact of drug absorption, especially in cases undergoing extensive bowel resection. Since previous studies about CAPS with bowel necrosis are mainly case reports, guidelines for the diagnosis and treatment of this condition are developed with “very low certainty of evidence.”^[9]^ We found that DOACs can be an effective secondary thromboprophylaxis for CAPS patient who had undergone massive bowel resection. Important clinical suggestions can be proposed from the clinical course of this case.

In terms of diagnosis, this is a young patient with a history of lower extremity deep venous thrombosis for more than 10 years who had been treated with vitamin K antagonists (VKAs) for 2 years and discontinued in the last 8 years. The risk factors of lower extremity deep venous thrombosis in this patient are obesity and occupation requires prolonged sedentary time, both have not been well controlled despite being monitored during 2 years of treatment. Moreover, during the previous 10 years, there was no diagnosis of APS and thus thromboprophylaxis was not strictly concerned. Conditions leading to this admission were acute manifestation of venous system occlusion: lower extremities veins in both legs, spleen veins, portal vein, superior mesenteric veins, renal, and superior mesenteric artery in 2 days which had caused life-threatening condition. According to the Sydney 2006 criteria for the diagnosis of antiphospholipid syndrome,^[10]^ the patient had met 2 criteria: clinical criteria: multiple venous thrombosis and arterial thrombosis and lab criteria: the presence of lupus anticoagulant in plasma. Differential diagnoses of positive lupus anticoagulant including systemic lupus erythematosus, autoimmune diseases, cancer or taking certain medications such as phenothiazines, penicillin, quinidine, hydralazine, procainamide, was excluded because the clinical manifestations of the patient were discordant to these conditions. Tests that help to identify systemic lupus erythematosus including antinuclear antibody, anti dsDNA, and SLE also yielded negative results.

Experts concluded that the treatment for CAPS is extremely difficult, of which the mortality rate is still as high as around 50% of cases.^[3,4]^ It is recommended to manage CAPS with anticoagulants, steroids, plasmapheresis, and/or intravenous immunoglobulins.^[11]^ For first-line treatment, best practice guidelines strongly recommend the use of therapeutic dose anticoagulation, especially unfractionated heparin, while other therapies are considered conditional recommendation.^[9]^ Glucocorticoid would benefit patients with concomitant active systemic lupus erythematosus, immune thrombocytopenia, or small vessel vasculitis; plasmapheresis is useful for cases with microangiopathic hemolytic anemia, while there is some evidence to use intravenous immunoglobulins patients with immune thrombocytopenia.^[9]^ Our patient was given emergency surgery to remove the necrotic bowel segments (Fig. 2) caused by superior mesenteric artery and vein occlusion. Following the first-line treatment from best practice guideline, we used low molecular weight heparin (Lovenox) 0.6 mL subcutaneous injection every 12 hours and switched to Acenocoumarol (Sintrom) when the patient discharged. At revisit, the patient condition was stable but INR did not reach the target value and was continuously unable to control after 3 follow-up revisits, so he was switched to dabigatran (Pradaxa) 110 mg twice daily or rivaroxaban (Xarelto) 15 mg per day. According to the 2018 European Heart Rhythm Association Practical Guideline, DOACs should be used with recommended dosage and the INR is unsuitable for the assessment of the anticoagulant activity of DOACs^[12]^ and therefore was not monitored during using DOACs. A meta-analysis in 2018 has shown that among APS patients treated with DOACs, most recurrent thromboses occur within 12 months after initial treatment, of which the risk of triple-positive patients is 4-fold higher than other subgroups.^[13]^ Our patient was positive for lupus anticoagulant only, and as no thrombosis event was observed in our patient during 24 months of follow-up, we suggest that DOACs is an effective and reasonable treatment for non-triple-positive APS.

**Figure 3:**
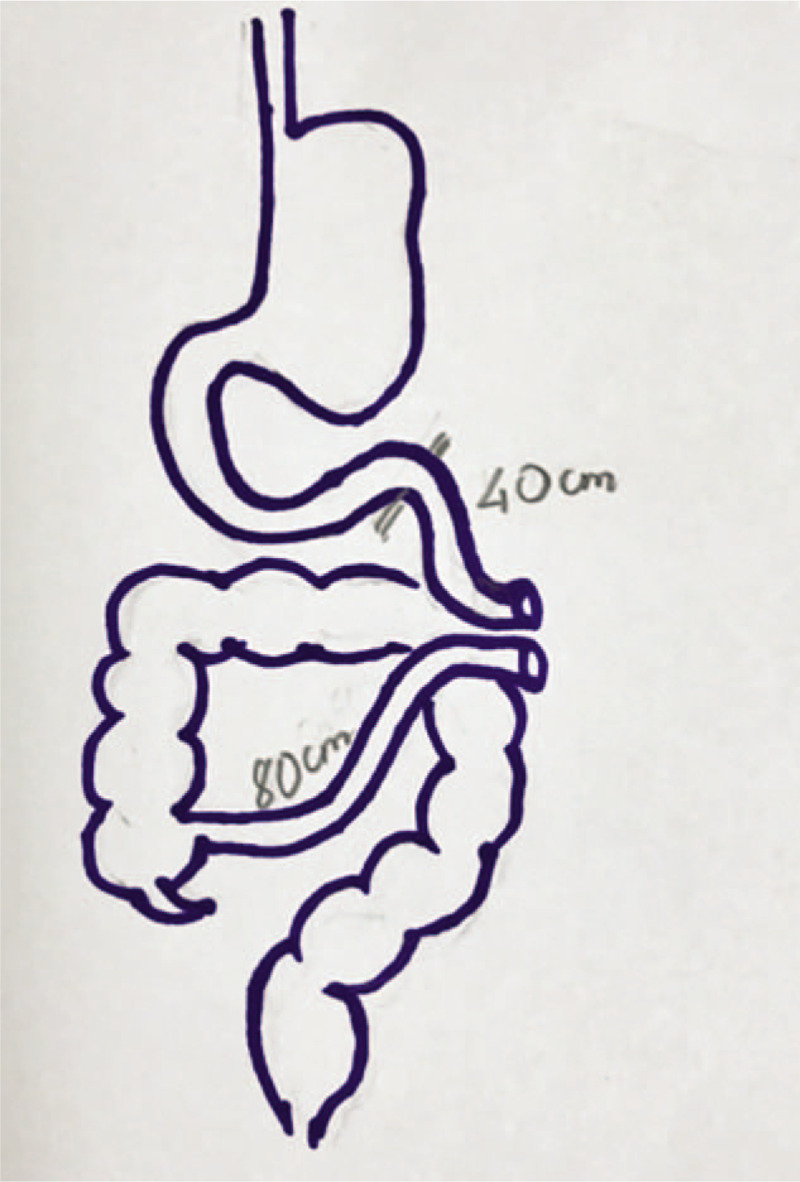
Demonstration of massive bowel resection: a total of 6 m small intestine was removed, the remaining small intestine was 40 cm after the duodenum and 80 cm before the ileocaecal valve.

Controversy has been raised when comparing the thromboprophylaxis efficacy and safety of DOACs and VKAs. Most studies had shown that rivaroxaban did not reach the noninferiority threshold to VKAs,^[14,15]^ therefore VKAs remained the first-line treatment for lifelong thromboprophylaxis in APS patients.^[1]^ However, since the risk for recurrent thrombosis was nonsignificant in those trials, authors suggested that DOACs could be considered an effective and safe alternative therapy for specific APS subgroups,^[1,13,14]^ as long as DOACs is avoided in triple-positive APS.^[16]^ The 2019 European League Against Rheumatism concluded that DOACs can be used to handle cases who are intolerant to vitamin K antagonists.^[1]^ Another factor that must be taken into consideration is in terms of pharmacokinetics, VKAs are absorbed primarily in the small intestine while DOACs such as dabigatran and rivaroxaban are absorbed primarily in the stomach.^[1]^ Our patient had undergone massive bowel resection including part of duodenum, jejunum, and ileum (Fig. 3), the absorption of VKAs is severely affected since the INR was continuously unable to be controlled after 3 revisits. In concordance with the case report from Lee ^[7]^ DOACs were chosen for our patient and have proved their efficiency during 24 months of follow-up.

In conclusion, CAPS is a rare, life-threatening condition of antiphospholipid syndrome, the diagnosis of which is often overlooked due to emergency situations of patients when hospitalized. It is important to pay attention to cases of multiple, recurrent occlusion to have early diagnosis and aggressive treatment that improve survival of patients. Several factors must be considered when prescribing secondary thromboprophylaxis for CAPS patients, particularly those who had undergone bowel resection. While VKAs is amongst the most important and fundamental treatment, physicians should be aware that VKAs are absorbed via the small intestine. For CAPS cases who are unable to absorb VKAs, DOACs is a reasonable alternative which has been found to be as safe and effective as VKAs in terms of thrombosis prevention.

## Author contributions

**Conceptualization:** Dinh Hieu Nhan, Suzanne Monivong Cheanh Beaupha

**Formal analysis:** Dinh Hieu Nhan, Suzanne Monivong Cheanh Beaupha

**Writing – original draft:** Dinh Hieu Nhan, Suzanne Monivong Cheanh Beaupha

**Writing – review & editing:** Dinh Hieu Nhan, Suzanne Monivong Cheanh Beaupha
